# A Real-Time PCR Method to Detect the Population Level of Halovirus SNJ1

**DOI:** 10.1371/journal.pone.0155642

**Published:** 2016-05-18

**Authors:** Yunjun Mei, Congcong He, Wei Deng, Dala Ba, Ming Yang, Jian Zhang, Shunxi Zhang, Ping Shen, Xiangdong Chen

**Affiliations:** 1 School of Chemical and Environmental Engineering, Wuhan Polytechnic University, Wuhan, Hubei, China; 2 State Key Laboratory of Virology, College of Life Science, Wuhan University, Wuhan, Hubei, China; CNR, ITALY

## Abstract

Although viruses of haloarchaea are the predominant predator in hypersaline ecosystem, the culture studies about halovirus-host systems are infancy. The main reason is the tradition methodology (plaque assay) for virus-host interaction depends on culturable and susceptible host. Actually, more than 90% of haloarchaea are unculturable. Therefore, it is necessary to establish an approach for detecting the dynamics of virus in hypersaline environment without culture. In this study, we report a convenient method to determine the dynamics of halovirus SNJ1 based on quantitative real-time PCR (qPCR). All findings showed that the qPCR method was specific (single peak in melt curves), accurate (a good linear relationship between the log of the PFU and the C_t_ values, R^2^ = 0.99), reproducible (low coefficient of variations, below 1%). Additionally, the physicochemical characteristics of the samples tested did not influence the stability of qPCR. Therefore, the qPCR method has the potential value in quantifying and surveying haloviruses in halophilic ecological system.

## Introduction

Haloarchaea inhabit hypersaline environments such as salt lakes, salterns and even saturated salt. Haloviruses, viruses of haloarchaea, are the predominant predators in hypersaline environments. Although some progress has been made on haloviruses, knowledge about haloviral ecology and halovirus-host ecosystems remains incomplete, resulting from the lack of *in vitro* halovirus culture systems [1, 2]. Porter et al. [3] proposed that traditional plaque assay at laboratory disposal was unable to completely reflect the actual facts in natural hypersaline waters. They emphasized that the dominant haloarchaeal members of hypersaline waters were now available, but it was not clear whether these dominant species were infected by the dominant haloviruses. Furthermore, virus infection is a complex process, depending on the three-component system of viruses, hosts and environmental factors. Any alteration in the three-component system would lead to a large discrepancy. However, the traditional plaque assay is unable to overcome these shortcomings. Therefore, it is necessary to establish a method to explore the interaction of haloviruses with their hosts in natural ecosystem independent of sensitive host cells. Recently, several studies have advanced our ability to observe such virus-host interaction at the single-cell level. Tadmor et al. [4] established a method by using microfluidic digital PCR to detect viruses in individual environmental bacteria; Moraru et al. [5] employed a GeneFISH technique for linking the presence of genes with cell identity in environmental samples; Kenzaka et al. [6] used an *in situ* DNA amplification technique to examine the propensity for phage-mediated gene transfer in freshwater environments at the single-cell level; and Allers et al. [7] revealed the single-cell dynamics of viral infection at the population level with phageFISH. These techniques represent the best available methods to provide experimental evidence for the interaction between an uncultivable phage and its host, and to visually display the entire process of single-phage infection. However, few methods are available to accurately display the population dynamics of viruses in extremely hypersaline ecosystems. Quantitative real-time PCR (qPCR), developed by Higuchi et al. [8], is a powerful tool and broadly used in many different areas for quantitatively analyzing the copy number of template DNA or RNA [9–12].

To establish an easy, sensitive and cost-effective method to detect the dynamics of viruses in hypersaline environments, we tested qPCR with a cultured host-virus system in order to determine its feasibility for use with environmental samples with the goal of ultimately investing the population dynamics of halovirus in a natural hypersaline habitat. Here, halovirus SNJ1 was taken as an example. SNJ1, a temperate and spherical haloarchaeal virus, was induced from the lysogenic host, *Natrinema* sp. J7-1, with mitomycin C, and the virus produced plaques on lawns of *Natrinema* sp. J7-2 [13, 14]. The infectious cycle of SNJ1 on J7-2 cells showed that the eclipse period was 3 h and the latent period was 4 h, and the burst size was 100–150 viruses per cell [13, 14].

## Materials and Methods

### Viruses, strains, media, and growth conditions

The haloarchaeal strains and viruses used in this study were listed in Table 1. *Natrinema* was incubated in halo-2 complex medium [15], *Haloferax* and *Haloarcula* were incubated using 18% MGM [16].

**Table 1:** Strains and virus used in this study.

No.	Species	Strains	Source
1	*Natrinema* sp.	J7-2	This laboratory
2	*Haloarcula hispanica*	ATCC 33960	Gifted by Kamekura M. Noda Institute for Scientific Research, Japan
3	*Haloferax vocanii*	WFD11	Gifted by Doolittle W.F. Dalhousie University, Canada
4	*Haloferax mediterranei*	ATCC 33500	Gifted by Meseguer I. University of Alicante, Spain
5	halovirus	SNJ1	This laboratory
6	halovirus	SNJ2	This laboratory

### Preparation of viral stock solution

Logarithmic-phase cultures of *Natrinema* sp. J7-2 were reinoculated into triangle flasks with 50 ml liquid Halo-2 medium at 2% v/v and aerobically incubated for 36 h at 37°C. Then, the cultures were centrifuged (13,523 *g* at 4°C for 6 min; Eppendorf, Hamburg, Germany), and infected with SNJ1 at a multiplicity of infection (MOI) of 10 at 37°C. At 1 h post-infection, the infected cells were collected by centrifugation as describe above, washed twice with Halo-2 liquid medium and re-inoculated into fresh pre-heated Halo-2 liquid medium and cultured at 37°C for 36 h with low aeration. Then, the cell debris was removed by centrifugation (13,523 *g* at 4°C for 15 min), yielding the viral stock solution that was stored at 4°C.

### Water samples

Six water samples (Table 2) were selected on the basis of physicochemical characteristics to test the influence of their properties on the efficacy of the qPCR methodology. Water samples W1 to W4 were taken from the underground brines in the Yunying Depression in the Jianghan Basin (Hubei, China), where *Natrinema* sp. J7-2 and *Natrinema* sp. J7-1 were obtained a few decades ago. Water samples W5 and W6 were taken from salt lake brine in Daban City (Sinkiang, China) and Dacaidan (Qinghai, China). Permissions for sampling were obtained from the local government, no specific permit was needed. These field study did not involve endangering to protected species.

**Table 2:** Characteristics of the underground brines.

No	pH	Ion concentration (g/L)					Organics (mg/L)
		Na^+^	Ca^2+^	Mg^2+^	Cl^-^	SO4^2-^	
W1	7.35±0.05	113.15±0.56	0.62±0.07	0.09±0.03	165.71±0.74	15.96±0.03	0.85±0.02
W2	7.52±0.1	115.37±0.25	0.71±0.03	0.10±0.01	167.20±0.65	15.38±0.05	0.76±0.05
W3	7.29±0.11	116.41±0.38	0.61±0.05	0.09±0.01	168.39±1.53	16.05±0.06	0.91±0.13
W4	7.41±0.08	108.96±0.24	0.65±0.03	0.09±0.01	159.85±1.48	15.18±0.05	0.65±0.09
W5	7.45±0.09	98.74±0.58	0.74±0.04	2.83±0.21	157.26±1.15	8.52±0.12	0.87±0.14
W6	8.64±0.1	32.57±1.25	0.1±0.01	43.16±0.45	163.50±0.83	28.36±0.53	1.24±0.86

### Primer design

Gene 23 encodes the ATPase of halovirus SNJ1, and the length of Gene 23 is 827 bp [14]. The primers amplifying a fragment of Gene 23 were designed using Primer Premier 5.0 software and synthesized by Invitrogen (Invitrogen, Shanghai, China). The length of the amplified product was 231 bp, and it had no homologous sequences in the chromosome of *Natrinema* sp. J7-2. The sequences of the forward primer and the reverse primer were 5'-ACGAGTGGCGGCAGGTTT-3' and 5'-TCCCGATGATGCGGTTGT-3', respectively.

### Treatment of samples with DNase I

DNase I endonuclease was used to remove the interference of uncoated phage DNA in samples. Each sample for qPCR was digested with DNase I (Biosharp, Anhui, China) without exception. The digestion mixture contained 2 μL DNase I (50 U/μL) and 98 μL viral stock, which was incubated at 37°C for 60 min and then at 99°C for 30 min to inactivate the enzyme, followed by cooling to room temperature. The digested solutions were stored at -20°C for later evaluation. All treated samples were performed in triplicate.

### Tests of the inclusivity and exclusivity of the qPCR

The inclusivity (i.e., detection of the target halovirus SNJ1) and exclusivity (i.e., non-detection of the non-target halovirus) of the qPCR were tested using three types of templates. The first template was consisted of halovirus SNJ1 and any one haloarchaeal strain listed in Table 1; the second template was composed of haloviruses SNJ1, SNJ2 and any one haloarchaeal strain as above; the third type only contained any one haloaracheal strains or halovirus liste in Table 1 except halovirus SNJ1. The diluted solution of halovirus SNJ1 as a control. And the final titer of SNJ1 in the first, the second and the control was identical. All haloarchaeal strains were listed in Table 1. And the final titer of SNJ1 in the first, the second and the control was identical. All haloarchaeal strains were listed in Table 1.

The qPCR conditions were optimized using real-time PCR kits (Tiangen, Beijing, China). Each reaction consisted of 20 μL solution, which included 7.6 μL deionized water, 0.4 μL ROX, 10 μL 2× TaqMasterMix (which included SYBR Green I), 1 μL template and 1 μL of each primer (5 μmol/L). Thermal cycling parameters were an initial 5 min at 95°C, followed by 40 cycles of 30 s at 94°C, 50 s at 60°C and 30 s at 72°C. The qPCR was executed with a 7500 Fast Real-Time PCR System (Applied Biosystems, Rotkreuz, Switzerland) using the default settings.

### qPCR of viral stock and statistical analyses

The viral stock solution was treated with DNase I as described above, and then the treated sample was serially diluted (10-fold, v/v) with deionized water for qPCR. Amplification experiments were executed using a 7500 Fast Real-Time PCR System as described above, and the protocol concluded with a melting curve program using 0.5°C increasing increments (10 s each; 60°C-94°C). Cycle threshold (C_t_) values, the number of PCR cycles required to amplify a target DNA to a detectable level, were calculated at a normalized fluorescence emission intensity threshold of △Rn = 0.1, which corresponded to approximately 5% of the maximum intensity. The standard curve was based on triplicate measurements.

For analyzing viral preparations amplified by qPCR, correlation coefficients (R^2^), mean C_t_ values and standard deviations (SDs) were calculated using origin 8.0 software. qPCR efficiency was calculated using the following formula: Efficiency = (10^−1/slope^ -1) ×100 [17–19]. To determine the precision of the qPCR assay, we calculated the coefficients of variation (CVs) for the Cts of all samples.

### Population level of SNJ1 determined by plaque assay and qPCR

For the population level of SNJ1 experiments, the early-logarithmic-phase culture of *Natrinema* sp. J7-2 was infected with SNJ1 at an MOI of 50 for 1.5 h; the infected cells were then collected (13,523 *g* at 4°C for 6 min; Eppendorf, Hamburg, Germany) and washed twice and re-inoculated into fresh, pre-warmed Halo-2medium at 37°C with low aeration. Samples were taken at the indicated intervals and centrifuged to remove cells and debris (13,523 *g*, at 4°C for 10 min). Subsequently, viral titers were determined by plaque assay and qPCR. The samples for qPCR were treated with DNase I as described above. All samples were treated in triplicate.

### Simulation of qPCR using water samples

To evaluate the feasibility of qPCR and the detection limit in environmental samples, the six water samples from the Yunying Depression, Daban City and Dacaidan were inoculated with viral stock solution and a viral titer of approximately 1×10^8^PFU/ml was obtained. The four inoculated water samples were then serially diluted (10-fold, v/v) with deionized water for qPCR. A standard curve of halovirus SNJ1 for each water sample was created by plotting the C_t_ values as a function of the titer of virus, using the standard curve from the qPCR of viral stock as a control. The statistical analyses were performed as above.

### Precision testing

To assess the precision of the qPCR method in a natural situation, viral stock solutions were diluted (10-fold, v/v) with the six water samples from the Yunying Depression. The diluted solutions were digested with DNase I as described above and then diluted with deionized water (100-fold, v/v) for qPCR. After amplification, intra- and inter-group CVs were analyzed. As a control, the viral stock solution was digested with DNase I and serially diluted (10-fold, v/v) up to 10^3^-fold with deionized water for qPCR.

## Results

### Tests of the inclusivity and exclusivity of the qPCR

The inclusivity and exclusivity of the qPCR were estimated. Among all templates tested, the qPCR was able to detect the amplification products from the first template, the second templates and the control, but not from the third type of template. These results indicate that the qPCR methodology is specific for halovirus SNJ1.Furthermore, the C_t_s of all template were nearly identical (the C_t_s between 21.36±0.06 and 20.97±0.09, respectively), which suggested that interference from other strains or halovirus was negligible.

### qPCR of viral stock and statistical analyses

**Fig 1:**
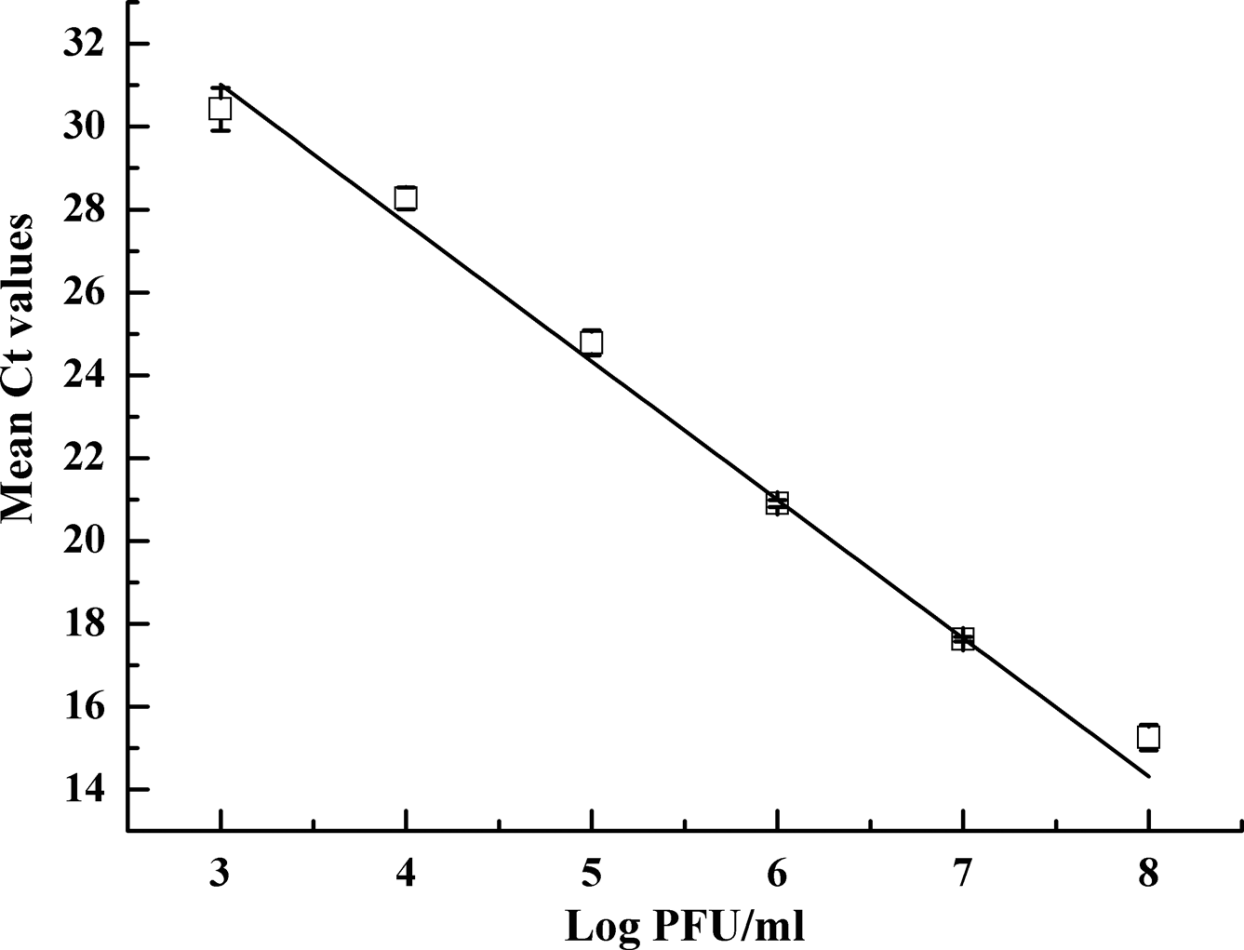
Standard curve for ten-fold serial dilutions of virus SNJ1. The C_t_ values are plotted against the corresponding log of the plaque-forming units per ml. R^2^ = 0.998; the amplification efficiency of qPCR is 99.27%.

**Fig 2:**
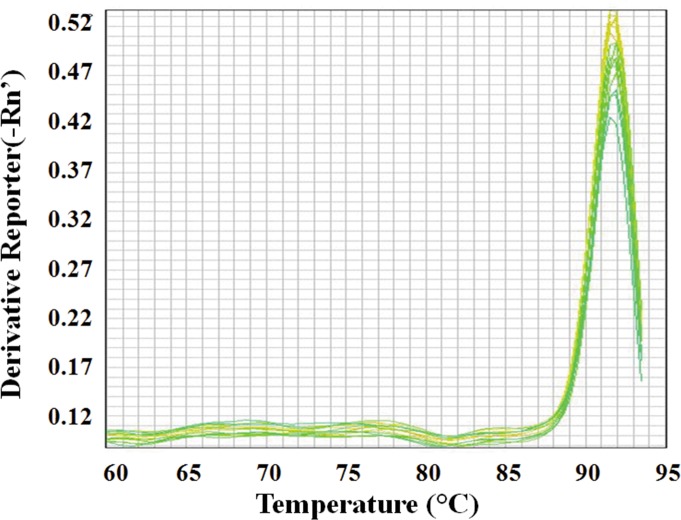
Melting curve analysis of qPCR products. The graph represents qPCR reactions plotted as the negative first derivative of the temperature versus fluorescence (-dRFU/dT) against temperature, representing a function of the decrease in SYBR Green I fluorescence due to increasing temperature.

The standard curve of halovirus SNJ1 was created by plotting the C_t_ values as a function of the PFU per ml (Fig 1). The linear regression equation was as follows: y = -3.34x + 41.03. A good linear relationship between the log of the PFU and the Ct values was obtained (R^2^ = 0.99). According to the amplification equation [(10^−1/slope^ -1) ×100], the slope was -3.34, and the amplification efficiency of qPCR was 99.27%. The CVs of the qPCR were low, varying from 0.40% to 2.03% over a seven-log range, which exemplified the reproducibility of the qPCR method. The melting curves showed only one peak, which suggested that the qPCR amplifications were specific (Fig 2). These findings indicated that the qPCR system was feasible and credible. All results indicated that the qPCR and plaque assay exhibited a good correlation.

### Population level of SNJ1 determined by plaque assay and qPCR

**Fig 3:**
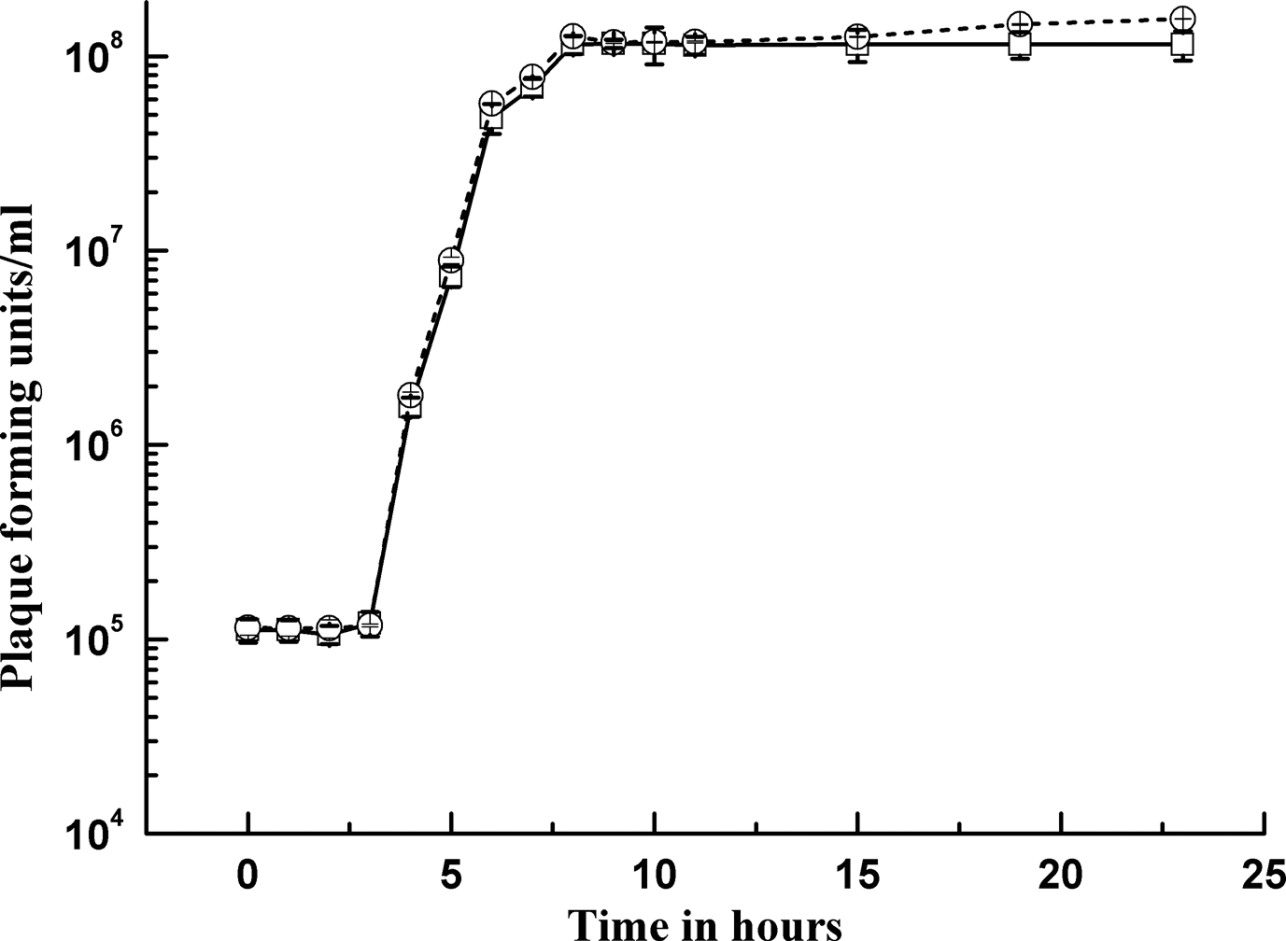
Population dynamics of SNJ1 determined by plaque assay and qPCR. The cells of *Natrinema* sp. J7-2 were infected by halovirus SNJ1 with an MOI of 50 for 1.5 h, then samples were taken from the culture system at indicated times, and the titers of samples were detected by plaque assay and qPCR. (○) viral titer determined by qPCR; (□) viral titer determined by plaque assay.

To evaluate the method of qPCR in measuring the population level of virus, we simulated viral dynamics by infecting *Natrinema* sp. J7-2 with SNJ1. The infected cells were broken subsequently, and the mature viral progenies were released. The titer of virus in the culture system changed during the time of infection, and it was determined by plaque assay and qPCR. The C_t_ values from the qPCR were converted to the titer of virus based on the standard curve of halovirus SNJ1. The result showed that viral alteration during the infection time as determined by qPCR dovetailed with the alteration indicated by the plaque assay (Fig 3), indicating that the qPCR was able to detect the population level of halovirus SNJ1 and the changes of halovirus SNJ1.

### Simulation of qPCR using water samples

**Fig 4:**
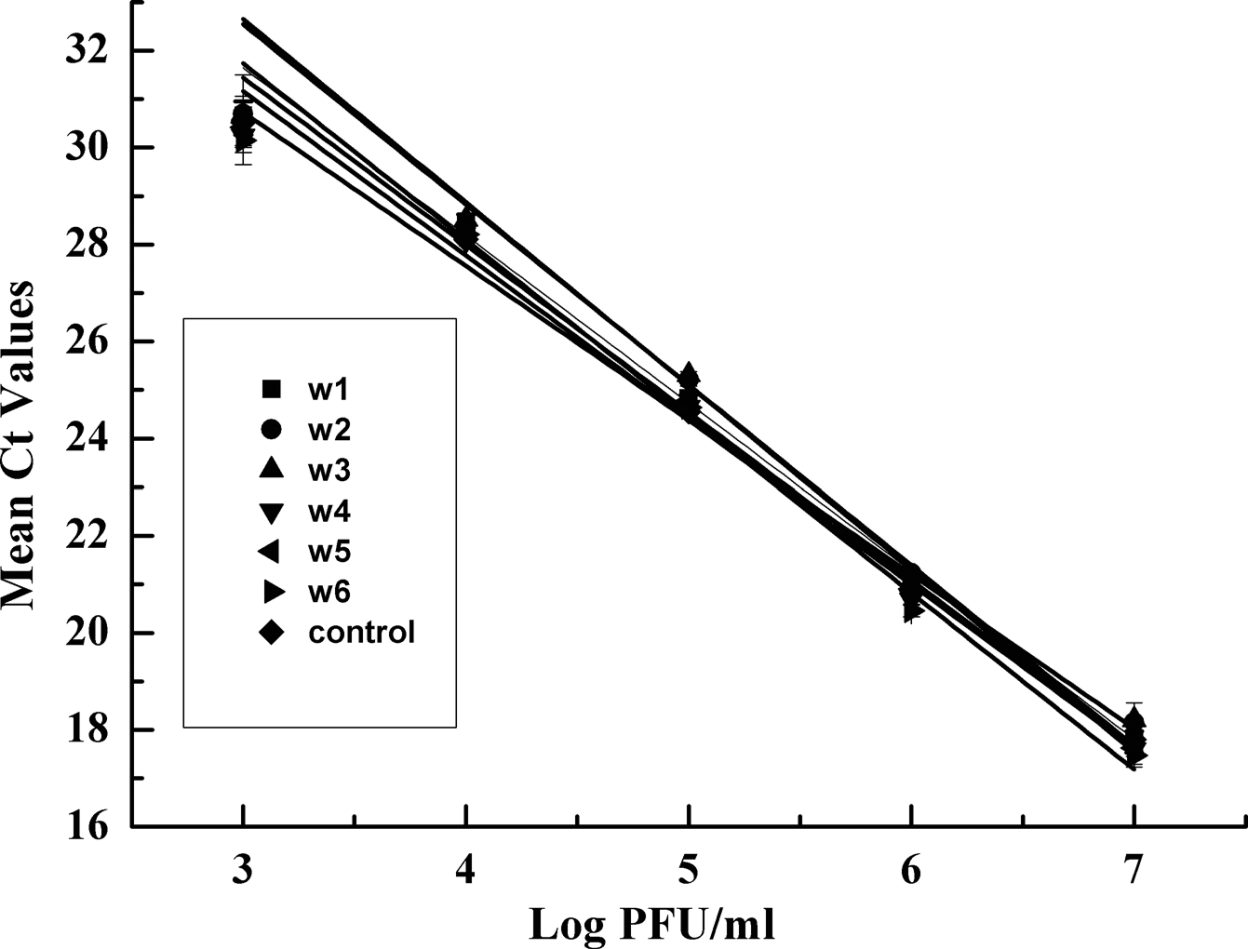
Standard curves for ten-fold serial dilutions of halovirus SNJ1 added to natural water samples. The C_t_ values are plotted against the corresponding log PFU/ml of halovirus SNJ1. R^2^ >0.98 and amplification efficiency was more than 83% in all cases.

Standard curves were created for each of the six water samples by plotting the C_t_ values as a function of the titer of virus (Fig 4). The results showed that a good linear relationship between the log of the titer of virus and the Ct values (R^2^ > 0.98 in all cases) was obtained in each case. The amplification efficiencies varied from 84.00% to 94.59% across the six qPCR assays. All these findings suggested that the physicochemical characteristics of the water samples tested had no obvious influence on the efficacy of qPCR because no significant differences were observed among the mean C_t_ values of the four water samples tested and of the viral stocks. Additionally, in all cases, the qPCR showed a high degree of sensitivity, with a detection limit of 1×10^3^ PFU/ml.

### Precision testing

In order to evaluate the stability of qPCR in other situations, the viral stock solution was treated as described above, and qPCR was performed to detect the titer of SNJ1. The results are listed in Table 3. The intra-group CVs varied from 0.19% to 0.78%, and the inter-group CV was 0.86%. These findings showed that the variance of tested samples was very low, which suggested that the physicochemical characteristics of the treated samples had no influence on the efficiency of qPCR. These experiments proved that the qPCR method was able to measure the viral titer accurately in the given conditions.

**Table 3:** The precision testing of quantitative real-time PCR.

Treatment	Average of Ct	CVs of intra-group	CVs of inter-group
W1	21.02±0.17	0.78%	0.86%
W2	21.23±0.04	0.19%	0.86%
W3	21.04±0.09	0.43%	0.86%
W4	20.68±0.1	0.48%	0.86%
W5	21.16±0.15	0.71%	0.86%
W6	20.98±0.12	0.57%	0.86%
Control	20.91±0.08	0.38%	0.86%

## Discussion

The traditional plaque assay is the gold standard for viral quantification and the investigation of virus-host interactions. This method depends on *in vitro*-culturable, sensitive host cells and viral ability to induce host cell lysis and produce plaques on lawns of a limited diversity of host cells [1, 2]. Thus, it is difficult to establish an *in vitro* system for detecting virus-host interactions. Furthermore, virus infection is a complex process, depending on the three-component system of viruses, hosts and environmental factors. Any alteration in the three-component system would lead to a large discrepancy. Therefore, it is necessary to establish a method independent of sensitive host cells. Here, we report a highly specific qPCR method for rapid quantification of halovirus in extreme hypersaline environments. It allows for a considerably easier, more sensitive and more cost-effective quantification of haloviruses and has the potential for wide application in routine surveys of the diversity and dynamics of haloviruses.

At the onset, we investigated the efficiency, reproducibility and specificity of our qPCR method and established the standard curve of the SNJ1 halovirus by plotting the C_t_ values as a function of the PFU. The linear regression of the scatter points between the log of the PFU and C_t_ values showed a good linear relationship, and we also observed high amplification efficiency, low CVs and specific qPCR amplification (Figs 1 and 2). To evaluate the ability of qPCR to detect the dynamics of a viral population, we combined qPCR with a plaque assay to monitor the titer of halovirus SNJ1 infecting *Natrinema* sp. J7-2. The titer determined by the two methods dovetailed except for some slight differences (Fig 4). All results indicated that the titer of halovirus SNJ1 employed here as determined by qPCR correlated well with the titer that was detected by plaque assay. The results of testing the feasibility of qPCR for amplifying halovirus SNJ1 in different environments also showed that the qPCR method was applicable in the tested situation.

Although qPCR-based methods are the most common techniques broadly applied for the sensitive detection and quantification of environmental microorganisms successful detection and quantification of the target organism require overcoming some common problems associated with environmental DNA-based methods [20] and developing an optimal microenvironment for PCR. Here, in order to ensure the stability, specificity and efficacy of qPCR, we investigated the following aspects.

To acquire a good correlation between the plaque assay and the qPCR method, we prepared a fresh viral stock solution, and all samples were treated with DNase I before qPCR amplification. The above treatments were necessary because the fresh viral solution guaranteed the infectivity of halovirus SNJ1, and the DNase I digestion removed the naked viral DNA. To overcome the effect of environmental factors on the qPCR, all samples were diluted with deionized water prior to qPCR. Our results showed that DNase I treatment was effective. Fortunately, we obtained high-quality viral DNA by diluting the viral stock solution with deionized water and omitting DNA extraction because halovirus SNJ1 was unable to endure low ionic strength [13], which differed from previous, related research [6,19], and this process reduced the experimental error caused by manipulation of DNA extraction. However, not all haloviruses are broken down when they are diluted with deionized water because a few of them are able to endure the low ionic strength [20]; therefore, DNA extraction is necessary for these haloviruses.

Haloviruses inhabit a complex saline environment, and samples obtained from such niches cannot be amplified by qPCR directly because the high ionic strength and other factors affect the efficiency of qPCR. Cordier et al. [17] proved that a dilution process was effective in reducing PCR inhibition caused by extraction contaminants in other organisms. As for our experiments, the deionized water not only ruptured viral particles but also reduced the ionic strength. Additionally, the amplification efficiency of qPCR was satisfactory (99.27%, Fig 1).

To evaluate the reliability of the qPCR method in detecting haloviruses in natural environments, we selected six environmental water samples to evaluate halovirus SNJ1 stock prior to qPCR, and the results showed that the influence of the natural environment was negligible. Although we have attained some achievements in quantifying halovirus SNJ1 using qPCR, further research work should be done to expand qPCR application range, such as other haloviruses or viruses in extreme, complex and diverse ecosystems. Of course, the qPCR method also has some defects, for example, it's based on the known DNA sequence of virus. But this problem would be solved gradually with the appearance of the metagenomic approach[21, 22] and single virus sequencing [23].
